# Interdependence of Anti-Inflammatory and Antioxidant Properties of Squalene–Implication for Cardiovascular Health

**DOI:** 10.3390/life11020103

**Published:** 2021-01-29

**Authors:** Nurul ‘Izzah Ibrahim, Isa Naina Mohamed

**Affiliations:** Pharmacoepidemiology and Drug Safety Unit, Department of Pharmacology, Faculty of Medicine, Universiti Kebangsaan Malaysia Medical Centre, Jalan Yaacob Latif, Bandar Tun Razak, Cheras, Kuala Lumpur 56000, Malaysia; nurulizzah@ukm.edu.my

**Keywords:** squalene, oxidative stress, antioxidant, anti-inflammatory, interdependence

## Abstract

Cardiovascular diseases (CVD) have been recognized as the leading cause of mortality worldwide, accounting for 31% of global mortality. Among the risk factors of CVD, hyperlipidemia has been established as the most potent risk factor. Statins, a class of drug that reduces lower-density lipoprotein cholesterol (LDL-C), are the preferred medical treatment. However, due to the development of statin-associated muscle symptoms, statins are associated with patients’ discontinuation and nonadherence. Other statin-induced side effects, such as hepatotoxicity and gastrointestinal upset, all contribute to patients choosing alternative medicines. Squalene (SQ), an unsaturated hydrocarbon naturally synthesized in plants and animals, could become the alternative treatment or supplementary agent for cardiovascular health. SQ has been shown to exert cardioprotective effect via its antioxidant activity. Oxidative stress and inflammatory responses are closely related to each other, which proposes an interdependence relation between antioxidant and anti-inflammatory. Therefore, this review explores the interdependence between the antioxidant and anti-inflammatory effects of SQ implicated on cardiovascular health.

## 1. Introduction

Cardiovascular diseases (CVD) have been recognized as the leading cause of mortality worldwide with approximately 17.9 million deaths per year [1]. CVD may refer to several linked pathologies that are commonly defined as coronary heart disease (CHD), cerebrovascular disease, peripheral arterial disease, rheumatic and congenital heart diseases and venous thromboembolism. CVD accounts for 31% of global mortality with the majority consisting of CHD and cerebrovascular accident [1,2]. An estimation by the World Health Organization (WHO) stated that 80% of premature CVD is preventable by controlling various risk factors to assist in reducing the increasing CVD burden on individuals and healthcare providers [3]. These risk factors include dyslipidemia, smoking, diabetes, hypertension, and family history of premature heart disease, as well as the non-independent risk variables of physical inactivity and body weight and composition [4]. In a well-recognized study, namely, the INTERHEART study, several CVD risk factors were highlighted, such as dyslipidemia, smoking, hypertension, diabetes, abdominal obesity, and it also demonstrated the protective effects of a healthy lifestyle (e.g., consumption of fruits and vegetables, and regular physical activity) [5]. Among all the risk factors, hyperlipidemia, which is defined as an abnormally elevated level of lipids or lipoproteins in the blood, has been established as the most potent risk factor [6,7]. It has been acknowledged that almost 50% of the general population have elevated cholesterol levels above the accepted normal range, corresponding with the prevalence of cardiovascular disease [8,9].

The first step to reduce cholesterol levels is lifestyle modification, including healthy diet, weight control, and physical activity, which are known to be effective. However, some individuals may find this difficult to achieve as there are limitations in most weight loss studies. For example, weight reduction might be able to reduce both cholesterol and triglyceride, but in a long-term period, almost half of the initial weight loss is regained after 1 year [10]. In a review by Makris and Foster (2011), the authors concluded that the type of diet is less significant due to the regaining of weight after the initial loss in a long-term period [11]. Due to this downside of lifestyle modification, it may be sensible to accomplish the lipid-lowering goals by preferably initiating medications more quickly rather than later. However, if lifestyle-change goals are achieved for long-term periods with no rebound, the demand for medication can be reconsidered.

In terms of pharmacotherapy, many studies have established that for most hyperlipidemia patients, statins, a class of drugs that reduce LDL-C are the preferred medical treatment [7,12,13,14]. However, despite their extensive use, statins are associated with patients’ discontinuation and nonadherence which creates a major gap in the prevention and treatment of atherosclerotic cardiovascular diseases. The main reason for the discontinuation is due to the development of statin-associated muscle symptoms [15]. It should be stated that the side effects occur more commonly at high doses and are more common in females, the elderly, patients with hypothyroidism and patients taking other drugs such as gemfibrozil and cytochrome P450 3A4 inhibitors [16]. Additionally, there is also a range of other statin-induced side effects, such as hepatotoxicity, gastrointestinal upset, as well as increased risk of getting cataracts and diabetes [15]. Due to the fear of the side effects, many people prefer complementary and alternative medicines to pharmaceutical products. This alternative is preferred as it is less expensive, requires no prescription and it is considered natural and safer. Several alternative treatments have been identified and discussed in several reviews which demonstrated efficacy in reducing plasma lipids with the use of garlic, artichoke leaf extract, fenugreek, red yeast rice, omega-3 fatty acids and plant stanols [17,18,19]. Other complementary and alternative medicines are constantly being discovered in providing alternatives to statin-intolerant patients.

Squalene (SQ), a highly unsaturated hydrocarbon from the triterpenoid family, is synthesized in plants, animals, bacteria and fungi as a precursor for the synthesis of secondary metabolites such as sterols, hormones, or vitamins [20,21]. SQ has been discovered as a major component of shark liver oil, which was believed by the Japanese people to possess powerful healing agents [22]. High concentration of SQ in the shark liver oil has been associated with protective effects against bacterial and fungal infections, particularly in eczema and dry skin lesions [23,24]. Besides being the main source from shark liver oil, which is limited by animal protection regulations [25], SQ is also extracted from plant sources such as olive oil, soybean oil, rice, wheat germ, grape seed oil, peanut, corn, and amaranth. Among the plant sources, olive oil is the only source that provides commercial SQ, although amaranth has the highest content of SQ [26]. SQ also possesses a protective effect on the skin against UV-induced radiation damage due to its high secretion in sebum, about 10–14% of lipids on sebum [27]. In addition, SQ has demonstrated its anti-cancer properties by inhibiting Ras oncoprotein farnesylation and restricting the transformation of 3-hydroxy-3-methylglutaryl-coenzyme A (HMG CoA) into mevalonate, which takes place in cholesterol biosynthesis pathway [28]. It has also been found that SQ exhibited a cardioprotective effect, similar to statin, via inhibition of HMG-CoA reductase in the cholesterol biosynthesis pathway. As described earlier, statin is the preferred medical treatment for reducing LDL-C; its mechanism of action is by inhibiting HMG-CoA reductase in the cholesterol biosynthesis pathway [29]. Specifically, the inhibition of HMG-CoA reductase activity occurs due to the enhanced SQ-derived cholesterol synthesis via negative feedback mechanism. In a randomized controlled trial conducted by Chan et al. (1996), it was demonstrated that SQ supplementation at the dose of 860 mg/day for 20 weeks in primary hypercholesterolemia patients had significantly decreased total cholesterol (TC) and low-density lipoprotein cholesterol (LDL-C) levels compared to the placebo group. Additionally, this SQ supplementation had also caused a reduction in triglyceride (TG) levels by 5.3% and increased the high-density lipoprotein cholesterol (HDL-C) level by 1.8% [30]. This human trial has shown that SQ can cause hypocholesterolemic activity, which in turns may reduce the risk of cardiovascular disease.

**Figure 1:**
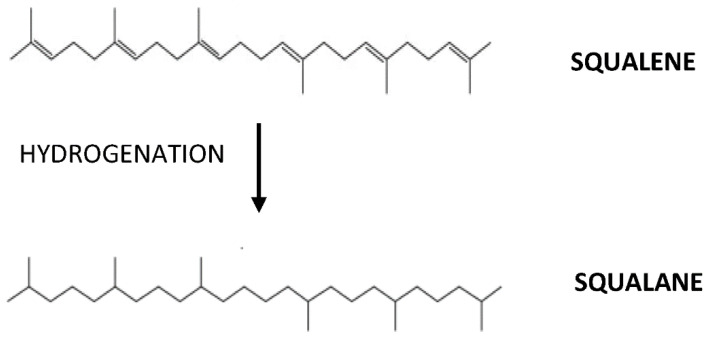
Following hydrogenation, squalene (C_30_H_50_) will develop into its oxidized form, squalane (C_30_H_62_).

In a systematic review by Ibrahim et al. (2020) [31], on the efficacy of SQ in cardiovascular diseases, it was concluded that SQ occupies cardioprotective effects from its antioxidant property. This property is associated with the abundance of double bonds in the structure, which has enabled SQ to act as a strong antioxidant [31]. The abundance of double bonds structure contributes to the extreme reactivity of SQ in getting into the oxidized form by binding with hydrogen ions from water releasing three unbound oxygen molecules and developing into its saturated form squalane, C_30_H_62_ (Figure 1). The released oxygen may then reach the cells to intensify cellular metabolism and improve the function of certain organs in the body [32]. However, the outcome of the review was heterogeneous, with 16 studies showing positive results on SQ supplementation in animals (*n* = 15) and humans (*n* = 1), while five studies showed inconsistent or negative results, with animals (*n* = 3) and humans (*n* = 2). These discrepancies of the SQ effect on cardiovascular diseases clearly warrant further investigation [31]. Indeed, oxidative stress, which is caused by exaggerated reactive oxygen species, is closely related to inflammatory responses and is interdependently related. The exact reason for the failure or inefficiency of an agent with antioxidant property is yet to be revealed. Many studies have supported that there is an interdependent relationship between inflammation and oxidative stress [33]. In a review by Lou-Bonafonte et al. (2018), it was stated that the anti-inflammatory and antioxidant properties of SQ are responsible for its various biological actions [34]. Nonetheless, the interdependence between these properties was not elaborated. Therefore, this article elucidates the effects of SQ on antioxidant and anti-inflammatory properties for an alternative mechanism of action, besides the antioxidant property alone and HMG-CoA reductase inhibition.

## 2. Antioxidant Activity of Squalene Related to Cardiovascular Health

Reactive oxygen species (ROS), which include free radicals, such as superoxide anion (O_2_^−^), lipid radicals (ROO·), hydroxyl radical (·OH), and non-radicals, such as hydrogen peroxide (H_2_O_2_), hypochlorous acid (HClO) and peroxynitrite (ONOO^−^), are the by-products of numerous oxidative physiological and biochemical processes. Under physiological conditions, ROS serve as signaling molecules that involve regulation of vascular smooth muscle cell contraction, relaxation, and growth [35]. On the other hand, pathophysiological conditions may provoke an imbalance between ROS (oxidants) and antioxidants, which leads to endothelial dysfunction and subsequent cardiovascular disease conditions [35,36]. ROS can exert direct oxidizing effects on DNA, proteins and lipids contributing to cell damage, necrosis, and apoptosis [36]. In the meantime, antioxidant, which can be defined as a substance when present at concentrations lower than the oxidizable substrate, may significantly decrease or prevent the adverse effects of reactive species [37]. Antioxidants are important for the defense mechanisms associated with free radicals’ attack. Therefore, the intake of natural-derived antioxidant has been suggested for preventing degenerative diseases caused by oxidative stress, including cancer, Alzheimer’s disease and atherosclerosis [38]. Due to these, many natural products have been tested for their antioxidant property using different assays [38]. Several previous studies have demonstrated antioxidant activity of SQ, with respect to cardiovascular health, using different methods such as lipid peroxidation, antioxidant enzymes and others, as tabulated in Table 1. In these studies, SQ has demonstrated its antioxidant effects in cardiovascular-related conditions including hyperlipidemia [39], atherosclerosis [40], myocardial infarction [41,42,43,44,45] and cardiotoxicity [46].

Gabas-Rivera et al. (2014), have measured the levels of ROS in isolated lipoprotein fractions including very low density lipoprotein (VLDL), low density lipoprotein (LDL) and high density lipoprotein (HDL). In the study, mice of the C57BL/6J strain have been used due to the higher predisposition to atherosclerosis development [39]. Three models of mice from this strain have been used, namely, wild-type, ApoE-deficient and ApoA1-deficient. ApoE and ApoA1 are examples of apolipoprotein, an important component of lipoprotein particles that facilitates the transport of cholesterol, TG and phospholipids between plasma and cells [47]. Therefore, mice that lacked both apolipoproteins have impaired elimination of lipoproteins, providing a possibility to explore changes in lipids [48]. In this study, the supplementation of 1 g/kg SQ for 11 weeks significantly increased plasma HDL-C level for all animal backgrounds, indicating that SQ exhibits an atheroprotective effect. It was also shown that SQ supplementation had significantly reduced the ROS level in LDL and HDL fractions for both wild-type and ApoA1-deficient mice. Meanwhile, SQ had demonstrated a significant reduction in ROS level for isolated VLDL and HDL in ApoE-deficient mice. This study has shown that SQ can reduce ROS levels in normal, ApoA1- and ApoE-deficient mice. In addition, plasma malondialdehyde (MDA), which is one of the most frequently used lipid peroxidation indicators, also showed significant reduction for all animal backgrounds following SQ supplementation [39]. This study indicates that SQ supplementation can produce antioxidant activity by reducing the oxidative stress level in lipoprotein fractions.

The SQ effect on antioxidant defenses was also studied by Guillén et al. (2008) via paraoxonase activity and plasma 8-isoprostaglandin F2α level. In this study, they categorized ApoE-knockout mice into two groups; male or female to investigate the relation of SQ administration modulation accordingly to sex-dependent manner. In terms of the atherosclerotic lesion, male mice receiving SQ showed a significant decrease in lesion area, while no change was observed in female mice indicating that SQ modulates lesion in a sex-specific manner. However, it was shown that SQ administration for 10 weeks via beverages did not induce significant changes in the activity of the paraoxonase in either sex [40]. Paraoxonase is an enzyme with anti-atherosclerotic property that generally inhibits the accumulation of lipoperoxides and inhibits the lipid oxidation of LDL [49]. In contrast, SQ has a significantly low lipid peroxidation in both sexes as observed via the measurement of prostaglandin namely 8-isoprostaglandin F2α [40]. The measurement of this prostaglandin, which was enhanced in cardiovascular risk factors, is a reliable method for identifying subjects with enhanced rates of lipid peroxidation [50].

Farvin and colleagues have performed several antioxidant experiments using SQ at 2% concentration, that was embedded in the animals’ standard diet for 45 days. The rats were injected for two days with isoproterenol to induce myocardial infarction (MI). The prior treatment of SQ had significantly reduced diagnostic marker enzymes [44], which demonstrates the cardioprotective effect of SQ. SQ supplementation has shown significantly increased activities of antioxidant enzymes (GPx and GST), as well as anti-peroxidative enzymes (CAT and SOD) in the MI-induced rats [45]. Subsequently, SQ has exhibited the ability to counteract lipid peroxidation in plasma and heart tissue [42,43,44]. The measurement of lipid peroxidation was conducted by means of malondialdehyde (MDA) via thiobarbituric acid (TBA) assay as the oxidative stress biomarker [42]. MDA is a commonly used oxidative stress biomarker in various health problems, including cardiovascular diseases, cancer, psychiatry and chronic obstructive pulmonary disease [51]. Farvin et al. had also shown that SQ had maintained glutathione (GSH) levels in the heart tissue at near-normal levels in isoproterenol-induced MI rats [43,44]. GSH is an antioxidant that prevents damage to cellular components against exogenous and endogenous toxins including reactive oxygen (ROS) and nitrogen (RNS) species [52]. The level of endogenous antioxidants, such as ascorbic acid and alpha tocopherol in the heart tissue, has also been measured, whereby the SQ administration has significantly reduced the isoproterenol-induced decline in these antioxidants level. Impairment of *α*-tocopherol status and inadequacy intake may cause damage to the cardiac muscles [53].

Dhandapani et al. (2007), conducted a study to determine the antioxidant status and lipid peroxidation of SQ and polyunsaturated fatty acids (PUFA) on isoproterenol MI-induced rats. In terms of cardiovascular-related results, it was found that SQ supplementation had significantly decreased the diagnostic marker enzymes such as alanine aminotransferase (ALT), alanine aminotransferase (AST), lactate dehydrogenase (LDH) and creatine phosphokinase (CPK). In terms of antioxidant and lipid peroxidation, the SQ-given group showed significantly reduced lipid peroxides and significantly elevated GSH and antioxidant enzymes including GPx, GST, CAT and SOD in the heart tissue compared to the negative control group. These results may indicate that SQ has exerted cardioprotection against isoproterenol-MI-induced changes.

Cardiovascular toxicity has been defined by the National Cancer Institute (NCI) as toxicity that affects the heart, including angina, acute arrhythmia, and myocardial infarction [54,55]. Most commonly, this type of toxicity is related to patients receiving chemotherapy (doxorubicin, anthracycline, cyclophosphamide) or targeted therapy (trastuzumab, bevacizumab and tyrosine kinase inhibitors) [55]. Although survival rates of cancer may have been improved due to advancement in chemotherapy and targeted therapies, the patients may however suffer from cardiac side effects. To overcome the cardiac side effects caused by certain drug administration, SQ has also been suggested. A study by Motawi et al. (2010), where SQ at 35 mg/kg body weight was orally supplemented to cyclophosphamide-induced rats has demonstrated a significant reduction in all cardiac markers, including CPK, LDH and AST. For antioxidant activity, the SQ treatment caused a significant decrease in GPx, while an increase in GSH level when compared to the cyclophosphamide-control group [46]. This study may indicate the protective effect of SQ via antioxidant capacity to attenuate the cardiotoxicity effects due to cyclophosphamide exposure.

**Figure 2:**
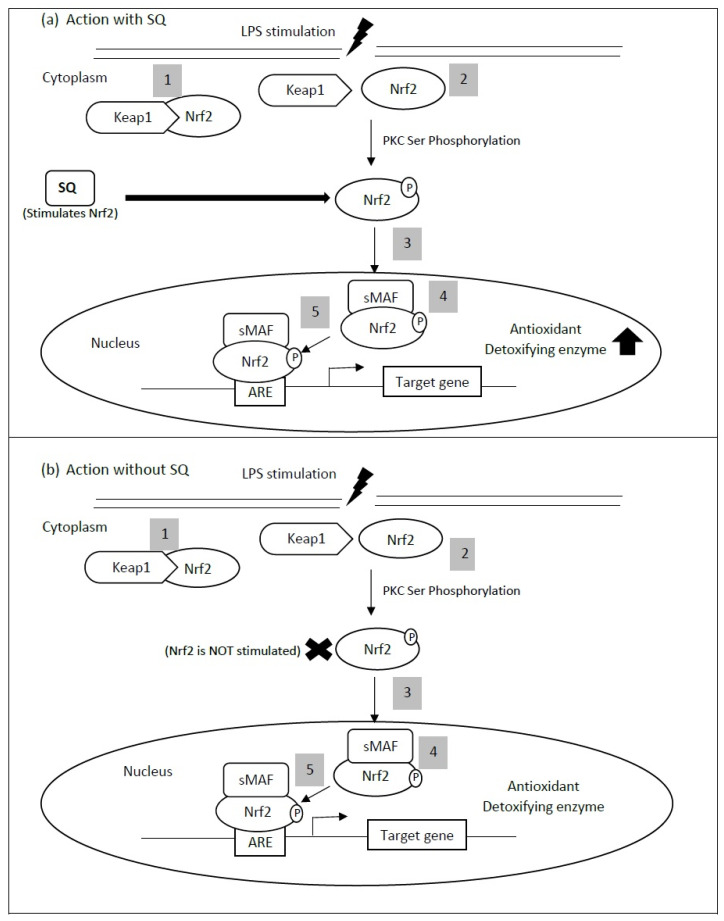
Keap1-Nrf2-ARE pathway. A proposed mechanism for the antioxidant property of squalene (SQ), where SQ stimulates the total and phosphorylated Nrf2. The stimulation of Nrf2, therefore, activates the transcription of antioxidant or detoxifying enzyme, which then causes a reduction in myocardial injury. (1) During normal conditions in the cytoplasm, Nrf2 resides as an inactive complex, with its repressor, Keap1. (2) Upon activation by oxidative stress (LPS stimulation), an oxidation of Keap1 cysteine residues or phosphorylation of Nrf2 serine (Ser) 40 may occur with respect to protein kinase C (PKC), which subsequently causes the release of Nrf2. (3) Nrf2 is then translocated into the nucleus and (4) dimerized with a small transcription factor, sMaf, forming a heterodimer. It then (5) binds to the antioxidant response element (ARE) genes. ARE is responsible for the regulation of antioxidant or detoxifying enzyme transcription. This figure is modified from Vomhof-DeKrey and Picklo, 2012 [58].

All studies mentioned above have demonstrated that SQ exerts a cardioprotective effect via its antioxidant activity. A transcription factor known as nuclear factor E2-related factor 2 (Nrf2) is accepted as a master regulator of antioxidant responses to cellular stress [56]. The SQ antioxidant activity might be related to this transcription factor, as SQ has been shown to significantly increase total and phosphorylated nuclear factor E2-related factor 2 (Nrf2) protein expression in lipopolysaccharide-treated cells (Figure 2) [57]. Nrf2 responds to oxidative stress by binding to antioxidant response element (ARE) in the promoter of genes coding for antioxidant enzymes [58]. Nrf2-regulated gene expression is mainly controlled by Kelch-like ECH-associated protein 1 (KEAP1) that mediates its protein ubiquitination and degradation. KEAP1, which acts as an adapter molecule for CUL-E3 ligase, will undergo cysteine modification upon exposure to oxidative stress triggering dissociation of KEAP1 from CUL-E3 ligase [59]. In addition, Nrf2 serine (Ser) 40 could be phosphorylated by protein kinase C (PKC) and dissociated from KEAP1 [58]. The altered KEAP1 structure causes the release of Nrf2, which is then translocated into the nucleus [60]. Upon entry into the nucleus, Nrf2 molecules will dimerize with other transcription factors, including small Maf (sMaf) forming a heterodimer, and bind to the ARE to induce gene transcription [61]. Nrf2 is involved in the induction of genes encoding many cytoprotective enzymes including heme oxygenase-1 (HO-1), glutamate cysteine ligase (GCL), NAD(P)H: quinone oxidoreductase-1 (NQO1), superoxide dismutase (SOD), glutathione S-transferase (GST), glutathione peroxidase (GPx) catalase (CAT), and thioredoxin [60,62]. Increased oxidative stress in the affected myocardium is a well-established phenomenon. Concerning heart failure, ROS causes impairment of cardiac function and increases arrhythmia risk by a direct toxic effect of increased cell necrosis and apoptosis [63,64]. Several Nrf2 downstream target genes, such as HO-1, SOD and GPx, have demonstrated protection against abnormal myocardial remodeling, pathological myocardial hypertrophy and heart failure [65,66,67]. Additionally, an overexpression of Nrf2 genes in the transverse aortic constriction mouse model of pressure overload has attenuated ROS production and hypertrophic growth in cardiomyocytes, and cardiac fibroblasts [68]. These previous studies may have indicated the protective effect of Nrf2 in cardiovascular health via reduction in oxidative stress.

## 3. Anti-Inflammatory Actions of Squalene

Inflammation plays an important role during the development of atherosclerosis, a dominant cause of cardiovascular disease. Initially, in response to oxidized LDL-C, injury or infection, resident or circulating leukocytes bind monocytes to the developing lesion site. As they resume to ingest chemically modified lipids and lipoproteins, monocytes turn into macrophages, which will be developed into foam cells, causing fatty streaks to be developed. The majority of the cells at the immediate plaque rupture site are macrophages, which indicates that they are the dominant type of atherosclerotic inflammatory cell infiltrates [69]. Other inflammatory mediators, including activated T cells and mast cells, are also present in the endothelium. Eventually, these inflammatory cells contribute to the development of the atheromatous lesion, which constitutes a lipid pool covered by a fibrous cap. The monocyte–macrophages release proteolytic enzymes, namely, metalloproteinases, which break down collagen in the fibrous cap, causing it to be susceptible to rupture. This event will expose the tissue factor and atherosclerotic debris beneath to arterial blood, which will then induce thrombosis. Smooth muscle cells in the arterial wall will be locally stimulated, which subsequently releases factors to recruit additional monocytes. This event may amplify the inflammatory response and promote a local procoagulant effect [70,71].

According to a study by Cardeno et al. (2015), SQ has shown significant potential in managing inflammatory conditions due to the overactivation of inflammatory cells such as monocytes, macrophages and neutrophils [57]. As described earlier, atherosclerosis, which is the initiator of most CVD, is associated with the overactivation of monocytes and macrophages. In the study, SQ has been shown to target pro- and anti-inflammatory mediators and pathways to modulate the over-activation of neutrophils, monocytes and macrophages. The inflammatory condition was induced using liposaccharide (LPS) on isolated murine macrophages from male Swiss mice and human monocytes as well as neutrophils from healthy subjects. LPS, which acts as an endotoxin, will bind to the CD14/TLR4/MD2 receptor complex that subsequently causes the secretion of pro-inflammatory cytokines, such as tumor necrosis factor alpha (TNF-α), interleukin (IL)-8, IL-6 or interferon gamma (IFN)-γ [72]. After binding with LPS, Toll/Interleukin-1 Receptor (TIR) of the TLR4 interacts with TIR of myeloid differentiation factor 88 (MyD88), which aggregates the signal and subsequently transmits to IL-1 receptor kinase (IRAK). In response to the signal transmission, IRAK will be phosphorylated and eventually activates the transcription factors, NF-κB [73]. LPS induction causes the modulation of several transcription factors including the peroxisome proliferator-activated receptor gamma (PPARγ), nuclear transcription factor (NF)-kB, nuclear factor-E2-related factor-2 (Nrf2) or mitogen-activated protein kinase (MAPK) family [74,75] via the increased level of several pro-inflammatory enzymes including inducible nitric oxide synthase (iNOS), cyclooxygenase-2 (COX)-2 or decreased level of antioxidant enzymes, such as haem oxygenase 1 (HO-1) [76,77]. During inflammation, matrix metalloproteinases (MMPs) are also induced and are important for regulating immune cell development, effector function, migration and ligand–receptor interactions, as well as activating signal transduction pathways [78]. Due to these modulations, the LPS-induced model is excellent for the screening and subsequent evaluation of potential drugs or natural products on the inflammatory pathway [79]. LPS induction causes endotoxemia, which is related to a significant increase in cardiac dysfunction [80], triggered via toll-like receptor 4 (TLR-4)-mediated inflammatory responses. The trigger leads to a chronic low-grade pro-inflammatory condition, namely, metabolic endotoxemia (ME), which is usually high in CVD patients [81] Following administration of SQ at 25 and 50 µM for 18 h, the in vitro study conducted by Cardeno et al. (2015), demonstrated attenuation in the inflammatory events caused by the LPS induction (Table 2) [57].

**Figure 3:**
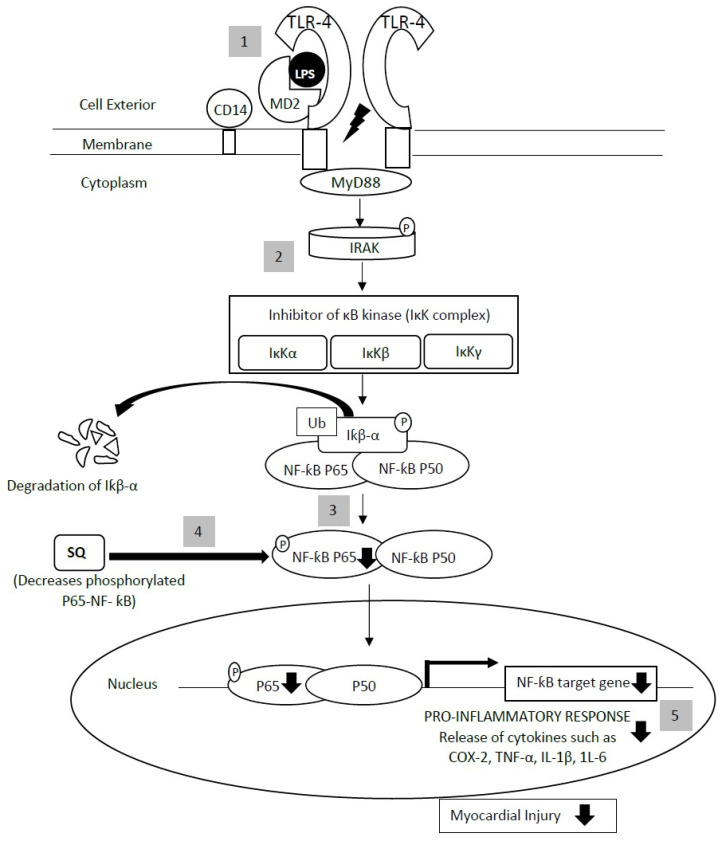
The effects of squalene in the NF-ƙB pathway. SQ decreases phosphorylated P65-NF-ƙB, which causes a reduction in pro-inflammatory response. (1) Upon stimulation of the LPS that binds to the CD14/TLR4/MD2 receptor complex, TLR4 interacts with MyD88, which aggregates the signal and is subsequently transmits to IRAK (becoming phosphorylated). (2) The phosphorylated IRAK then causes the activation of the transcription factors, NF-κB, via phosphorylation of IκBα by the IKK complex, which triggers its ubiquitination and proteasomal degradation. (3) NFκB is then free to translocate to the nucleus for inducing NF-κB target gene expression. (4) SQ decreases phosphorylated P65-NF-ƙB, which eventually (5) causes a reduction in pro-inflammatory responses, athus decreases the myocardial-related injury. LPS: lipopolysaccharide; MD2: myeloid differentiation factor 2; CD14: cluster of differentiation 14; TLR-4: Toll-like receptor-4; MyD88: myeloid differentiation factor; IRAK: IL-1 receptor kinase; IκK: inhibitor of κB kinase; Iƙβ-α: inhibitor of κB alpha; COX-2: cyclooxygenase-2; TNF- α: tumor necrosis factor-2; IL-1β: interleukin 1β; IL-6: interleukin 6; Ub: ubiquitination; P: phosphorylated. This figure is modified from Shih et al. (2018) [87].

SQ significantly reduced the protein expression of iNOS and COX-2 in the LPS-treated murine peritoneal macrophages [57]. In addition, SQ had also caused a significant reduction of phosphorylated P65-NF-KB while significantly increasing IKBα (Figure 3) [57]. Generally, the signaling pathways mediating NF-κB activation occurs through canonical (classical) or noncanonical (alternative) pathways, depending on the phosphorylation-induced ubiquitination of IκB proteins [82]. In resting state, NFκB is bound and inhibited by IκB proteins that conceal the nuclear localization signals, blocking their nuclear import [83]. For the canonical pathway, activation may occur within minutes of exposure to proinflammatory signals by LPS, growth factors, and antigen receptors, which will converge to an IκB kinase (IKK) complex, composed of catalytic (IKKα and IKKβ) and regulatory (IKKγ) subunits. Upon activation, IKK phosphorylates IκBα at two N-terminal serines, triggering its ubiquitination and proteasomal degradation. NFκB is, therefore, free to translocate to the nucleus with other transcription factors for inducing gene expression [82]. On the other hand, the noncanonical pathway is mediated by NFκB inhibitory kinase (NIK), activating IKK alpha, and directly acts on the non-IκB substrates of the NF-κB subunits to modulate the transcriptional responses [84,85]. SQ causes a modulation in phosphorylated P65-NF-κB and IKBα, which indicates that SQ modulates NFκB signaling system via the canonical pathway. Additionally, a previous study by Felices et al. (2019), also supported that SQ could alleviate LPS effect via NF-kB inactivation [86].

The transcription factor NFκB has been identified as a key player in the crosstalk between inflammation and cardiovascular diseases [88]. Previous studies have shown that the transcription factor NFκB is involved in the regulation of inflammatory cytokines, activation of genes involved in various cardiovascular diseases and pathogenesis of cardiac remodeling and heart failure [89,90]. In cardiac remodeling, the sustained activation of NFκB has been shown to be cytotoxic and contributes to heart failure via the triggering of a chronic inflammatory response [91]. Other than that, the activation of NFκB in the heart occurs in many conditions, such as acute ischemia and reperfusion [92,93], as well as in unstable angina [93,94]. These previous studies have indicated that NFκB is involved in the development and progression of cardiovascular diseases. Therefore, the ability of SQ to modulate NFκB signaling pathways may directly affect the pathogenesis of cardiovascular diseases.

In the study by Cardeno et al. (2015) [57], SQ demonstrated a significant reduction in mRNA levels of NFκB downstream genes such as TNFα and IL-1β in LPS-treated murine peritoneal macrophages [57]. During chronic response to myocardial infarction, an extended increase in the cytokine production may develop interstitial fibrosis and deposition of collagen in the non-infarcted zone leading to ventricle dysfunction, thus contributing to a deleterious effect on the cardiovascular system [95,96]. The ability of SQ to reduce the genes of proinflammatory cytokines, therefore, may curb the development of fibrosis and collagen leading to ventricle dysfunction. Meanwhile, in the LPS-treated human leukocytes incubated with 50 µM SQ, a significant downregulation of Toll-like receptor 4 (TLR-4) gene expression was demonstrated [57], which induced the release of pro-inflammatory and immunoregulatory cytokines through ligand stimulation [97]. LPS-induced myocardial inflammation is primarily transmitted by TLR4, whereby the binding to the respective ligand may further activate the NFκB pathway, therefore causing the activation of various inflammatory cytokine expression [98]. TLR4-mediated inflammation plays a role in cardiovascular disease, as it has been shown elevated in coronary atherosclerotic plaques [99,100].

SQ also had markedly attenuated LPS-mediated c-Jun-NH (2)-terminal kinase (JNK), but not P38 MAPK expression in murine peritoneal macrophages [57]. This finding was supported by a previous study that demonstrated opposing effects on heart failure development when chronic treatment with P38 MAPK and JNK inhibitors was given to a dilated cardiomyopathy (DCM) hamster heart [101]. This finding indicates the ability of SQ in attenuating JNK, a signaling pathway responsible for regulating cell fate implicated in multiple diseases, including neurological and immunological or inflammatory conditions [102].

In Cardeno et al. (2015), SQ downregulated the gene expression of metalloproteinases (MMPs) and upregulated peroxisome proliferator-activated receptor-gamma (PPAR ɣ) in LPS-treated human monocytes and neutrophils [57]. In atherosclerosis development, MMPs, especially MMP-9, contribute to cellular matrix degradation, which subsequently leads to the rupture of atherosclerotic plaques. On the other hand, PPAR*γ* that is commonly found in both vascular smooth muscle cells and macrophages, decreases the MMP expression and inhibits the vascular smooth muscle cells’ migration, thereby preventing plaque rupture [98,103]. Therefore, the ability of SQ to downregulate MMPs while upregulating PPAR*γ* can provide a beneficial effect to cardiovascular health, especially in atherosclerosis.

## 4. Interdependence of Anti-Inflammatory and Antioxidant Properties of Squalene in Cardiovascular Health

**Figure 4:**
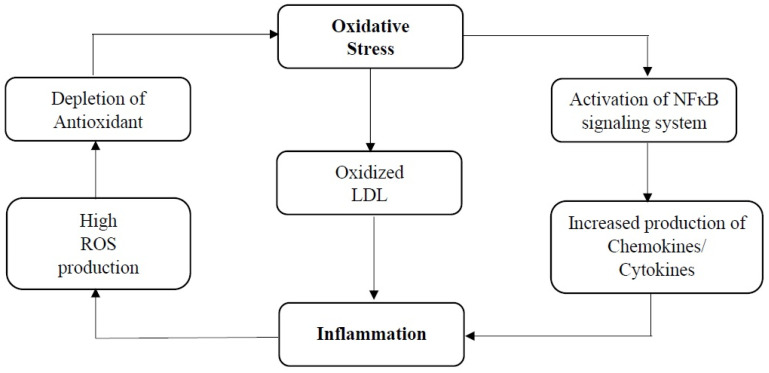
Interdependent relationship between inflammation and oxidative stress in cardiovascular-related diseases is caused by oxidized LDL. LDL particles, which are prone to oxidation, will become oxidized LDL under conditions of oxidative stress, which stimulates the production of chemokines, cytokines, and adhesion molecules, as well as activation and proliferation of lymphocytes, causing the occurrence of inflammation. Additionally, the activation of NF-ƙB may also result in immune cell activation, adhesion, and infiltration. In turn, inflammation causes oxidative stress as the production of the ROS is an essential property of activated immune cells. Therefore, oxidative stress and inflammation are involved in a self-propagating cycle. LDL: low-density lipoprotein; ROS: reactive oxygen species; NF-ƙB: nuclear factor kappa-B. This figure is modified from Vaziri (2008) [106].

Inflammation and oxidative stress are tightly interconnected with one another in cardiovascular health (Figure 4). For instance, atherosclerosis, the dominant cause of cardiovascular diseases, is closely associated with inflammation and oxidative stress that possess a potent role in atheroma formation. Stimulation of pro-inflammatory signaling pathways, expression of cytokine or chemokine, and increased oxidative stress are among the mechanisms leading to atherosclerosis [104]. At the site of inflammation, inflammatory cells, such as leukocytes and macrophages, liberate reactive species (reactive oxygen/nitrogen) that leads to exaggerated oxidative stress [33]. On the other hand, the oxidative stress activates the NFκB pathway that enhances proinflammatory gene expression [105,106]. These events will subsequently stimulate the release of chemokines, cytokines and adhesion molecules, as well as the activation of immune cells. Therefore, oxidative stress and inflammation are implicated in a self-propagating cycle [106]. Numerous studies had demonstrated the interdependent relationship of low-grade chronic inflammation and oxidative stress in chronic conditions of cardiovascular diseases [106,107].

Particularly for atherosclerosis, the pathogenesis is complex whereby it is commonly linked with the accumulation of low-density lipoprotein cholesterol (LDL-C) in the intima layer that subsequently undergoes modification by exposure to ROS, becoming an oxidized form of LDL-C [108]. The resulting oxidized LDL-C (OxLDL) may alter cellular permeability and gradually affect the arterial walls via activation of the innate immune system [109]. Indeed, the events of infiltration and retention of LDL-C in the intima layer have initiated an inflammatory response in the artery wall [110]. Along with several other factors such as shear stress and various cytokines, OxLDL also enhances endothelial expression of adhesion molecules such as E-selectin and vascular cell adhesion molecule 1 (VCAM-1), which can activate endothelium. This event has set up the basis for an increased expression of adhesion molecules and inflammatory genes, as well as endothelial dysfunction, which is an early marker for atherosclerosis [111,112]. Based on the pathogenesis of atherosclerosis, it was shown that inflammation and oxidative stress that took place in the event were inseparably interconnected. Therefore, it is important to identify the potential agent that acts interdependently to combat diseases with an interdependent relationship between inflammation and oxidative stress successfully.

**Figure 5:**
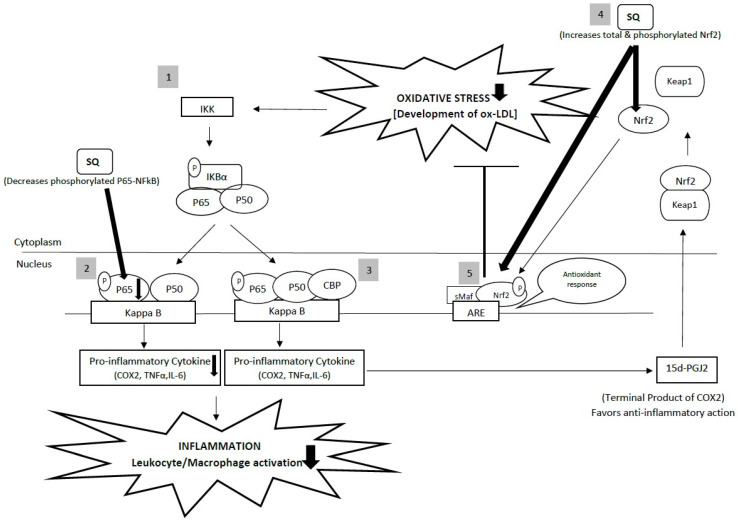
Summary figure describing the crosstalk between Nrf2 and NF-κB. (1) Activation of IKK complex due to oxidative stress, which in turn phosphorylates NF-kB, leads to its translocation into the nucleus. In the nucleus, activation of proinflammatory cytokines such as COX-2, TNF-α, and IL-6 occurs, upregulating the inflammatory process. (2) SQ decreases the phosphorylated P65- NF-ƙB that subsequently leads to the downregulation of inflammatory process. (3) NF-kB combines with the competitive Nrf2 transcriptional co-activator CBP producing a terminal product, 15d-PGJ2. This molecule acts as an inducer of Nrf2, which reacts with KEAP1, causing the release of Nrf2 from KEAP1. (4) SQ increases total and phosphorylated Nrf2, which dimerize with a small Maf (sMaf) forming a heterodimer, ultimately binding to the ARE to induce gene transcription. (5) Activation of the Nrf2-Smaf-ARE combination activates the target gene expression for an antioxidant response, which ultimately leads to the suppression of oxidative stress. This figure is modified from Ahmed et al. (2017) [116].

Anti-inflammatory and antioxidant can be linked via two key transcription factors, namely, NFκB and nuclear factor erythroid 2-related factor 2 (Nrf2), which regulate cellular responses to inflammation and oxidative stress, respectively [113]. NFκB is a family of transcription factors that consists of five members: p50, p52, p65 (Rel-A), c-Rel, and Rel-B proteins [113]. These molecules can be configured as homo- or heterodimers and persist as an inactive complex with the inhibitory molecules known as IκB proteins in resting cells. The activation of the NFκB signaling system occurs through canonical or noncanonical pathways [114]. As described earlier, SQ has been proposed to modulate NFκB signaling system via the canonical pathway, due to its ability to significantly reduce phosphorylated P65-NF-KB while significantly increasing IKBα [57]. Meanwhile, SQ has modulated Nrf2, which is a key transcription factor that regulates a network of antioxidant and cytoprotective genes by increasing the total and phosphorylated Nrf2 protein expression in LPS-induced macrophage [57]. NF-κB and Nrf2 pathways are interconnected in such crosstalk as depicted in (Figure 5). Oxidative stress will cause the activation to IKK, which in turn phosphorylates NF-κB, leading to its translocation into the nucleus. Upon arrival in the nucleus, NF-κB binds with Nrf2 co-activator CBP causing the transcription of pro-inflammatory cytokines, such as COX-2. The terminal product of COX-2, 15d-PGJ2 has been associated with anti-inflammatory effect in the early phase of inflammatory reaction, which may act as an inducer of Nrf2 reacting with KEAP1 and ultimately binding to antioxidant response elements (ARE) [115,116]. As a result, this binding causes the suppression of oxidative stress that is associated with cardiac and vascular abnormalities in many cardiovascular diseases.

Additionally, SQ had also upregulated one of the Nrf2 downstream target genes, HO-1 in human monocyte and neutrophil [57]. HO-1 is highly inducible with respect to various stimuli to protect cells against oxidative and inflammatory damage [117]. In the atherosclerosis-related study, HO-1 expression in macrophages demonstrated increased antioxidant protection and decreased inflammatory components of atherosclerotic lesions [118]. Interestingly, the effects of anti-atherogenic agents, including statin, have been shown to be mediated through HO-1 induction [119]. HO-1 catalyzes the oxidation of heme to generate carbon monoxide (CO), biliverdin, and iron, which have important antioxidant and anti-inflammatory properties, resulting in a vascular anti-atherogenic effect. The endogenously produced CO can act as a second messenger during cellular inflammation, proliferation, and apoptosis. Biliverdin, which will be reduced to bilirubin, has antioxidant properties. Meanwhile, ferrous iron causes the induction of ferritin expression, which is essential for the sequestration of iron [117].

## 5. Possible Application of Squalene Supplementation in Humans

**Figure 6:**
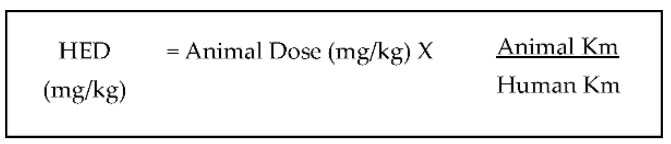
Formula for conversion method from animal to human dosage, based on body surface area (BSA). HED: human extrapolation dose; Km: Km factor. This formula is obtained from Reagan-Shaw et al. (2008) [122].

When considered as a treatment for lipid-lowering drugs, a 500 mg/day of SQ may be beneficial, as shown by a human trial by Miettinen and Vanhanen 1994 study. In the study, the 500 mg/day SQ dose for 6 weeks had significantly reduced intermediate-density lipoprotein cholesterol (IDL-C), triglycerides and phospholipids without increasing total cholesterol in the hypercholesterolemic patients. However, a long-term supplementation of 1000 mg/d SQ for 9 weeks did not show the same efficacy as a significant increase in total cholesterol was observed compared to the control group [120]. In a study by Strandberg et al. (1990), 900 mg/day SQ was supplemented to patients with cerebrovascular and cardiovascular disease with hypercholesterolemia for 30 days, which demonstrated inconsistent elevation of free and esterified cholesterol in the serum of the patients [121]. This study may indicate that SQ supplementation can increase cholesterol synthesis with an inconsistent pattern. Therefore, these human studies have shown an inconsistent result at the doses of 900 and 1000 mg/day but have demonstrated a reduction in lipid parameters at a lower dose of 500 mg/day. Conversely, in another human study by Chan et al. (1996), SQ supplemented at the dose of 860 mg/day for 20 weeks had significantly decreased TC and LDL-C levels compared to the placebo group. In addition to this human study, it was shown that an effective dose for cholesterollowering effect could range from 500 mg/day to 860 mg/day. In the study conducted by Chan et al. (1996), the authors revealed that the SQ dosage (860 mg/day) was chosen based on the standard recommendation by the manufacturer and may not be ideal for maximum cost-effectiveness [30]. Ideally, the determination of dose in human studies, especially for phase I and phase II clinical trials, should be based on the data from animal studies using an appropriate conversion method such as the body surface area (BSA) normalization method (Figure 6) [122]. The estimation of starting dose in clinical studies from animal dose should not be extrapolated using a simple conversion method based only on body weight. Generally, the BSA method has shown a good correlation among species for parameters such as oxygen utilization, basal metabolic rate, caloric expenditure, blood volume and circulating plasma protein. In addition, a safety factor should be considered for determination of high dose in animal toxicology study [123].

## 6. Conclusions and Future Perspectives

This review has condensed the antioxidant and anti-inflammatory properties of SQ. In cardiovascular health, the antioxidant properties of SQ are more pronounced compared to the anti-inflammatory properties. Interestingly, SQ has demonstrated its cardioprotective effect via the interconnection inflammatory pathway in the proposed mechanism of NF-κB and Nrf2 pathways. Indeed, SQ has a huge potential to become a nutraceutical supplement due to its interdependence on the stated properties. Further studies need to be conducted to determine whether these mechanisms (NF-κB and Nrf2 pathways) are indeed interconnected for SQ to exert its action of cardioprotection. Studies for SQ supplementation in animal studies could also be translated into human studies by conducting an updated randomized controlled trial using dose extrapolation from animal doses to find the appropriate dose for human application, especially for cardiovascular health.

## Figures and Tables

**Table 1:** Antioxidant effects of squalene in cardiovascular-related conditions.

Assays for Determining Antioxidant Activity	Cardiovascular-Related Conditions	Study Type	Experimental Model	Findings	Reference
Paraoxonase	Hyperlipidemia	Animal	Wild-type, ApoA1- and ApoE-deficient C57BL/6J mice	Reduction in reactive oxygen species (ROS) level and plasma malondialdehyde in lipoprotein fractions independently of the animal background.	[39]
Paraoxonase	Atherosclerosis	Animal	Female and male ApoE knockout mice	No significant changes in paraoxonase activity in both sexes.	[40]
8-isoprostaglandin F2α				Decreased level of plasma 8-isoprostaglandin F2α in both sexes.	
Catalase (CAT) and superoxide dismutase (SOD)	Myocardial infarction (MI)	Animal	Isopreterenol MI-induced Wistar male rats	Increased CAT and SOD activities. Increased GPX and GST activities.	[41,45]
Glutathione peroxidase (GPX) and Glutathione-S-Transferase (GST)					
Glutathione (GSH)				Increased GSH.	[41,43,44]
Thiobarbituric Acid (TBARS)				Decreased lipid peroxidation in plasma and heart tissue.	[41,42,43,44]
GPx and GSH	Cardiotoxicity	Animal	Cyclophosphomide- induced cardiotoxicity in male Wistar rats	Increased GSH and decreased GPx.	[46]

**Table 2:** Summary of SQ effects that attenuate inflammatory events caused by LPS induction.

Cardiovascular-Related Conditions	Study Type	Experimental Model	Findings	Reference
Atherosclerosis	In vitro	LPS-treated murine peritoneal macrophages	Suppression of iNOS and COX-2 protein expression. Significantly decreased phosphorylated JNK, but not p38 MAPK expression. Significantly decreased phosphorylated P65-NFκB, but significantly increase in IκB-α. Reduced mRNA levels of NFκB downstream genes including TNF- α and IL-1β.	[57]
		LPS-treated human monocytes	Significantly downregulated TLR-4, iNOS and COX-2 gene expression. Significantly reduced pro-inflammatory cytokine genes, TNF-α and IL-1β, but not IL-6 or IL-10. Significantly downregulated MPO and upregulated anti-inflammatory gene HO-1. Significantly down-regulated MMP-1 and MMP-9 gene expression. Significantly upregulated PPARγ gene expression.	
		LPS-treated human neutrophils	Significantly downregulated TLR-4 and iNOS gene expression. Significantly reduced pro-inflammatory cytokine genes TNF-α, IL-1β, IL-6 and IFN-γ. Significantly downregulated MPO and upregulated anti-inflammatory gene HO-1. Significantly down-regulated MMP-1 and MMP-3 gene expression. Significantly upregulated PPARγ gene expression.	

## Data Availability

Not applicable.
